# Gastric Cancer Surgery in an Emergency Setting: Insights from a Western Multicenter Cohort

**DOI:** 10.3390/jcm15166395

**Published:** 2026-08-19

**Authors:** Carlo Alberto Schena, Savino Occhionorelli, Vito Laterza, Caterina Cina, Filomena Misuriello, Domenico Lacavalla, Pierre Mathieu, Rocco Stano, Dario Andreotti, Giuseppe Quero, Claudio Fiorillo, Sergio Alfieri, Nicola de’Angelis, Fausto Rosa

**Affiliations:** 1 Department of General Surgery, Fondazione Poliambulanza, 25124 Brescia, Italy; 2 Unit of Emergency and Trauma Surgery, Sant’Anna University Hospital, 44124 Ferrara, Italy; 3 Department of Translational Medicine, University of Ferrara, 44121 Ferrara, Italy; 4 Department of Digestive Surgical Oncology and Liver Transplantation, University Hospital of Besançon, 25030 Besancon, France; 5 Emergency and Trauma Surgery, Fondazione Policlinico Universitario A. Gemelli IRCCS, 00168 Rome, Italy; 6 Digestive Surgery, Fondazione Policlinico Universitario A. Gemelli IRCCS, 00168 Rome, Italy; 7 Facoltà di Medicina e Chirurgia, Università Cattolica del Sacro Cuore, 00168 Rome, Italy

**Keywords:** gastric cancer, gastric surgery, emergency surgery, western cohort, morbidity, perforation, obstruction, hemorrhage

## Abstract

**Background**: Gastric cancer (GC) remains a leading cause of cancer death worldwide. A subset of patients presents acutely with life-threatening complications such as perforation, obstruction, or bleeding, requiring surgery under suboptimal conditions. This multicenter Italian study aimed to explore outcomes of emergency GC surgery, identify prognostic factors, and compare survival across clinical presentations. **Methods**: Between 2013 and 2022, 263 GC patients presented emergently at two high-volume Italian centers. Among them, 90 patients with histologically proven adenocarcinoma who underwent emergency surgery were included. Primary outcomes were 30-day mortality and overall survival (OS). **Results**: Obstruction was the most frequent presentation (41.1%), followed by hemorrhage (34.4%) and perforation (24.4%). Severe complications (Clavien–Dindo ≥ 3) occurred in 28.8% of patients, and thirty-day mortality reached 13.3%. Median OS was 6.5 months. Diffuse-type GC predicted shorter OS than intestinal-type (5.5 vs. 14 months; *p* = 0.015). Radical surgery was performed in 63.3% of cases and was associated with superior OS compared with palliative procedures (14 vs. 4.5 months; *p* < 0.001). The clinical presentation did not influence survival. On exploratory multivariable analysis, advanced age and diffuse histology were associated with higher 30-day mortality, while gastric resection was associated with better OS. **Conclusions**: Emergency GC surgery carries high morbidity and mortality. Age, histology, and gastric resection were independent prognostic factors, whereas clinical presentation did not significantly affect survival. Early diagnosis and tailored perioperative strategies are needed to improve outcomes.

## 1. Introduction

Gastric cancer (GC) ranks fifth in incidence and fourth in cancer-related mortality globally [1,2,3]. Despite advances in screening and multimodal treatment, a subset of patients still presents with acute, life-threatening complications necessitating emergency surgery. The management of such emergencies—typically perforation, obstruction, or hemorrhage [4,5,6]—is heterogeneous: perforation generally requires urgent source control, whereas hemorrhage and obstruction may, in selected cases, be managed by endoscopic, radiological, or temporizing/palliative strategies. When surgery cannot be avoided, it carries particularly high risks due to the absence of preoperative optimization, advanced disease stage, and physiological instability, posing unique challenges for surgical management and influencing both perioperative outcomes and long-term survival [4,7,8]. Consequently, outcomes reported from emergency surgical cohorts reflect both the biology of advanced disease and the selection of patients in whom immediate surgery is considered necessary.

Existing literature, mainly from Asia, suggests that emergency GC surgery is associated with increased morbidity and mortality compared to elective procedures [4,6,7,8,9]. Several studies have attempted to elucidate the specific challenges and prognostic implications of emergency GC surgery. These investigations have identified factors like advanced disease stage, inadequate lymph node dissection, positive surgical margins, and limited access to neoadjuvant therapy as potential contributors to poorer outcomes [9,10]. However, differences in tumor biology, comorbidity burden, and healthcare delivery between Eastern and Western populations limit the generalizability of these findings. Western evidence remains limited, mostly derived from small series or heterogeneous datasets, and a comprehensive understanding of factors influencing poor outcomes and optimal management strategies for emergency GC remains elusive.

This exploratory study had the primary aim of evaluating 30-day mortality and overall survival (OS) after emergency GC surgery. We explored three factors that could influence survival, namely the type of acute presentation (perforation, obstruction, hemorrhage); the histological subtype (intestinal vs. diffuse); and the surgical strategy (radical vs. palliative).

Our study provides one of the largest multicenter Western series of emergency GC surgery, aiming to complement existing literature and generate hypotheses for clinical practice.

## 2. Materials and Methods

### 2.1. Study Design and Patient Selection

This retrospective, multicenter study included patients undergoing emergency surgery for histologically proven GC at two academic hospitals (Fondazione Policlinico “A. Gemelli” IRCCS—Rome; Sant’Anna University Hospital—Ferrara) from 1 January 2013, to 31 December 2022.

A total of 263 patients with GC presented acutely to the emergency department. Patients treated conservatively or stabilized for semi-elective surgery were excluded (156 patients). One hundred and seven underwent emergency surgery. Four patients were excluded due to unknown survival, follow-up, or postoperative data, and 13 were excluded due to non-adenocarcinoma histology. Ninety patients with histologically confirmed gastric adenocarcinoma were included in the analysis (a flow chart is provided as Supplementary Figure S1).

The study was conducted in accordance with the ethical principles ascertained in the Declaration of Helsinki and approved by the Local Ethical Committee and the Institutional Review Boards (Fondazione Policlinico Universitario “A. Gemelli” IRCCS, Rome, Italy, ID: 5121/2022; Sant’Anna University Hospital in Ferrara, Italy, ID: CE: 597/2024/Oss/AOUFe). The clinical records of the eligible patients were retrospectively collected from a prospectively maintained database. In order to ensure consistent patient identification, the International Classification of Disease, 9th Revision, Clinical Modification (ICD–9CM) codes, which remain the standard hospital coding system in Italy, were used as follows: 151.1–151.9 (malignant neoplasm of the stomach) either as a primary or secondary diagnosis alongside 531.10–0, 532.10–50, 533.10–50, 534.10–50, 569.83 (gastrointestinal perforation), 567.0, 567.2, 567.8 (peritonitis), 531.31, 531.71, 531.91, 534.31, 534.71, 534.91, 560.89, 560.9 (gastrointestinal obstruction), or 531.0, 531.4, 534.0, 534.4, 578.0 (gastrointestinal hemorrhage). Clinical data were handled in accordance with the approved study protocols and the applicable institutional requirements for protection of patient information. Data collection, variable definition, and statistical methods followed the STROBE guidelines [11].

### 2.2. Study Variables and Definitions

The following demographic and clinical data were collected: age; sex; BMI; neoadjuvant therapy; type of emergency presentation (perforation, obstruction, hemorrhage); surgical unit type; type of surgical treatment; 30-day morbidity, described according to the Clavien–Dindo classification [12]; 30-day mortality; length of hospital stay [LOS]; initiation of adjuvant chemotherapy; overall survival (OS); histological type; resection margins status; lymph nodes status; and TNM stage.

Patients were operated on either by acute care surgical (ACS) units or by upper gastrointestinal (UGI) surgical units according to the availability of surgical theater and personnel. Regardless of their unit of affiliation, all surgeons were proficient in performing complex emergency surgery and oncologically radical gastric resections.

Radical surgery was defined as subtotal or total gastrectomy with curative intent and lymphadenectomy.

Palliative surgery included gastrojejunostomy, gastrostomy, jejunostomy, bypass procedures, or non-radical resections. Emergency gastrectomy for obstruction was performed only in cases of acute complete gastric obstruction with rapid clinical deterioration where bypass or stenting was not feasible. For hemorrhage, surgery was reserved for cases of uncontrolled bleeding refractory to endoscopic or interventional radiology techniques. In patients presenting with perforation, a procedure was classified as radical only when an oncologic gastrectomy with lymphadenectomy was performed with curative intent; procedures performed primarily for source control without curative intent were classified as non-radical/palliative. When histological subtype was known before the emergency event, as for patients already receiving oncologic treatment, the immediate emergency strategy remained primarily determined by the nature of the complication, the patient’s physiological condition, and oncologic resectability.

LOS was calculated from the time of surgery to discharge or death. Mortality was defined as any death occurring within 30 days after hospital admission. The histopathological type was classified according to the Lauren classification [13,14,15]. Resection margins were considered involved (or positive) if characterized by cancer cells at the proximal or distal edge of the surgical specimen. TNM staging was defined according to the 8th edition of the American Joint Committee on Cancer (AJCC) and the Union for International Cancer Control (UICC) [16,17,18].

### 2.3. Statistical Analysis

Patients were divided into different groups according to the histological type (intestinal vs. diffuse type), surgical unit type (acute care surgery vs. upper gastrointestinal surgery unit), diagnosis at the emergency department admission (perforation vs. obstruction vs. hemorrhage), and surgical setting (radical vs. palliative strategy). For the descriptive analysis, continuous variables were tested for normality with the Shapiro–Wilk test and were expressed as means and standard deviations (SD) in case of normal distribution, or as median and interquartile range (IQ) in case of skewed distributions. Categorical variables were expressed as counts and percentages (%). Categorical variables were analyzed using Fisher’s exact test, while normally distributed continuous variables were compared using the two-sample *t*-test and ANOVA. Non-normally distributed continuous variables were analyzed with the Mann–Whitney U test or Kruskal–Wallis test. OS was analyzed with the Kaplan–Meier method and compared between groups using the log-rank (Mantel–Cox) test. Multivariable logistic regression analysis was performed to explore factors associated with 30-day mortality, while Cox proportional hazards regression analysis was used to explore factors associated with OS. Given the limited number of 30-day deaths, the logistic regression analysis was considered exploratory, and its estimates were interpreted cautiously. Covariates for multivariable analyses were selected a priori according to clinical relevance and availability across the full cohort rather than solely on the basis of univariable statistical significance. A *p*-value < 0.05 was considered statistically significant. The statistical analysis was performed using R version 4.3.2.

## 3. Results

### 3.1. Study Population

Baseline characteristics of the 90 patients included in the study are reported in Table 1. The mean age was 69.7 years (SD: 12.6), and 40% were female. Median BMI was 23.73 kg/m^2^ (IQR: 21.22–25.96). Twenty percent of patients underwent emergency surgery while on neoadjuvant therapy. The most frequent presentation was obstruction (41.1%), followed by hemorrhage (34.4%) and perforation (24.4%). Radical surgery was performed in 57 patients (63.3%), most commonly total gastrectomy (38.9%). All surgeries were performed via an open approach. Positive resection margins were found in 11.1% of patients undergoing radical surgery, and at least 16 lymph nodes were harvested in 75.4% of cases. Complications occurred in 98.9% of patients, with severe complications in 28.8% of cases. Thirty-day mortality was 13.3%. Adjuvant chemotherapy was initiated in 34.4% of patients; median OS was 6.5 months (IQR: 3–19).

### 3.2. Factors Associated with the Histological Type

Fifty-six patients (62.2%) had diffuse-type GC, and 34 (37.8%) had intestinal-type GC. Age showed no difference between groups, whereas female sex was prevalent in the diffuse-type group. Compared with the diffuse type, intestinal type was significantly associated with radical surgical treatment (88.2% vs. 46.4%, *p* < 0.001), longer LOS (15 days vs. 11 days, *p* = 0.01), and higher OS (14 months vs. 5.5 months; *p* = 0.015). Diffuse type was significantly associated with a greater proportion of positive surgical margins (29.6% vs. 6.7%; *p* = 0.03) and a higher number of positive lymph nodes (4.5 vs. 2; *p* = 0.016). No significant difference in terms of 30-day mortality, death at follow-up, and number of retrieved lymph nodes was found between the two histopathological types. These results are summarized in the Supplementary Table S1.

**Figure 1:**
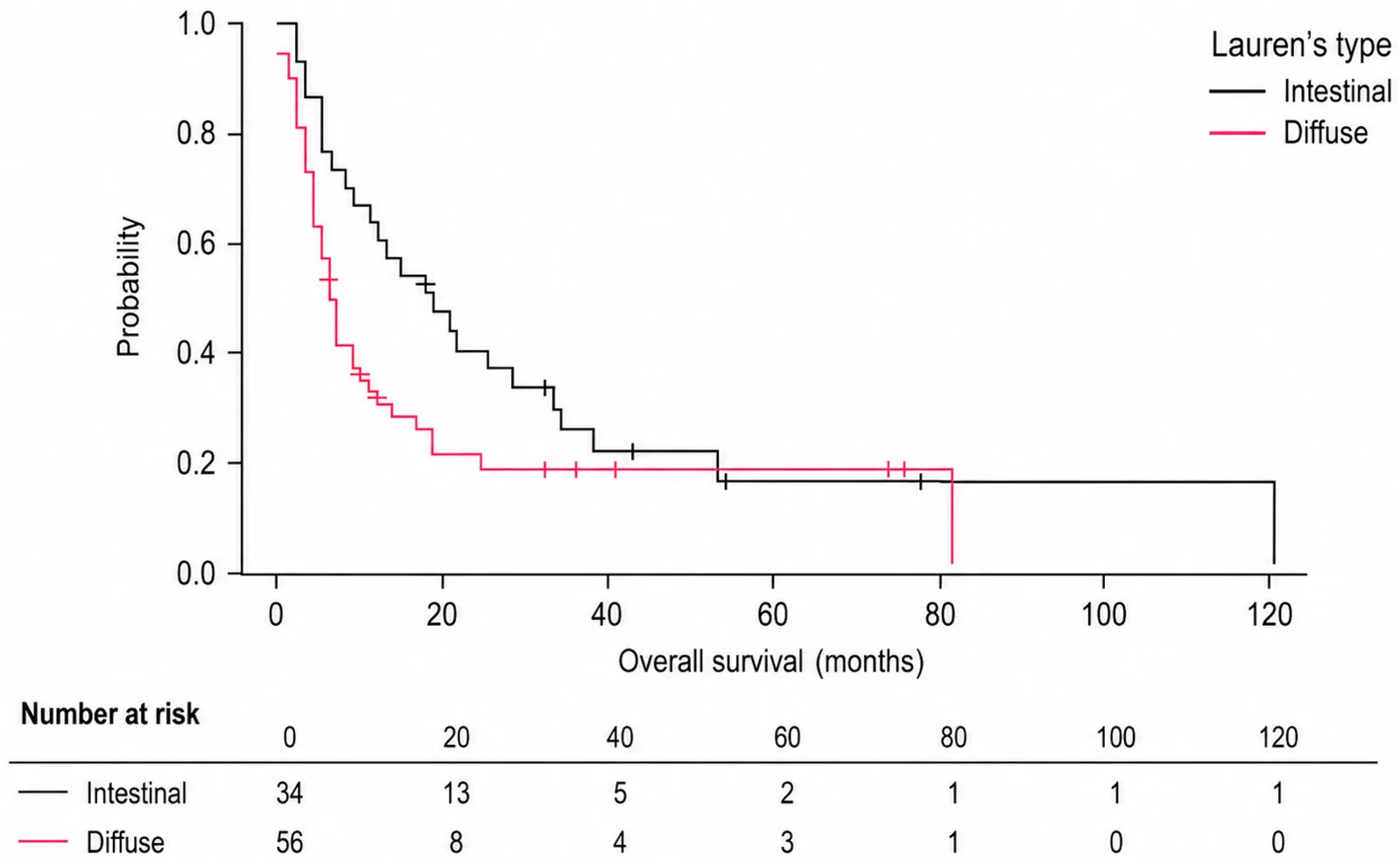
Kaplan–Meier curve of OS according to the histological type (black line: intestinal type; red line: diffuse type; *p* = 0.015).

Kaplan–Meier survival analysis (Figure 1) confirmed a statistically significant shorter OS for patients with diffuse-type histology compared to those with intestinal-type histology (*p* = 0.015).

### 3.3. Factors Associated with the Emergency Presentation

A statistically significant difference in age was observed among patients grouped according to emergency presentation (obstruction, hemorrhage, or perforation) (*p* = 0.002). Post hoc analysis with Bonferroni correction revealed that patients undergoing surgery for hemorrhage were significantly older than those with gastrointestinal occlusion (75.9 years vs. 65.4 years, *p* = 0.0014). There was no statistically significant association between the type of presentation and sex (*p* = 0.46), BMI (*p* = 0.32), neoadjuvant therapy (*p* = 0.1), length of hospital stay (*p* = 0.1), 30-day mortality (*p* = 0.57), number of positive lymph nodes (*p* = 0.33), presence of retrieved lymph nodes ≥ 16 (*p* = 0.28), positive surgical margins (*p* = 0.1), and adjuvant therapy (*p* = 0.34), and OS (*p* = 0.98). There was also no association between diagnosis at admission and death at follow-up (*p* = 0.58). These results are summarized in the Supplementary Table S2.

**Figure 2:**
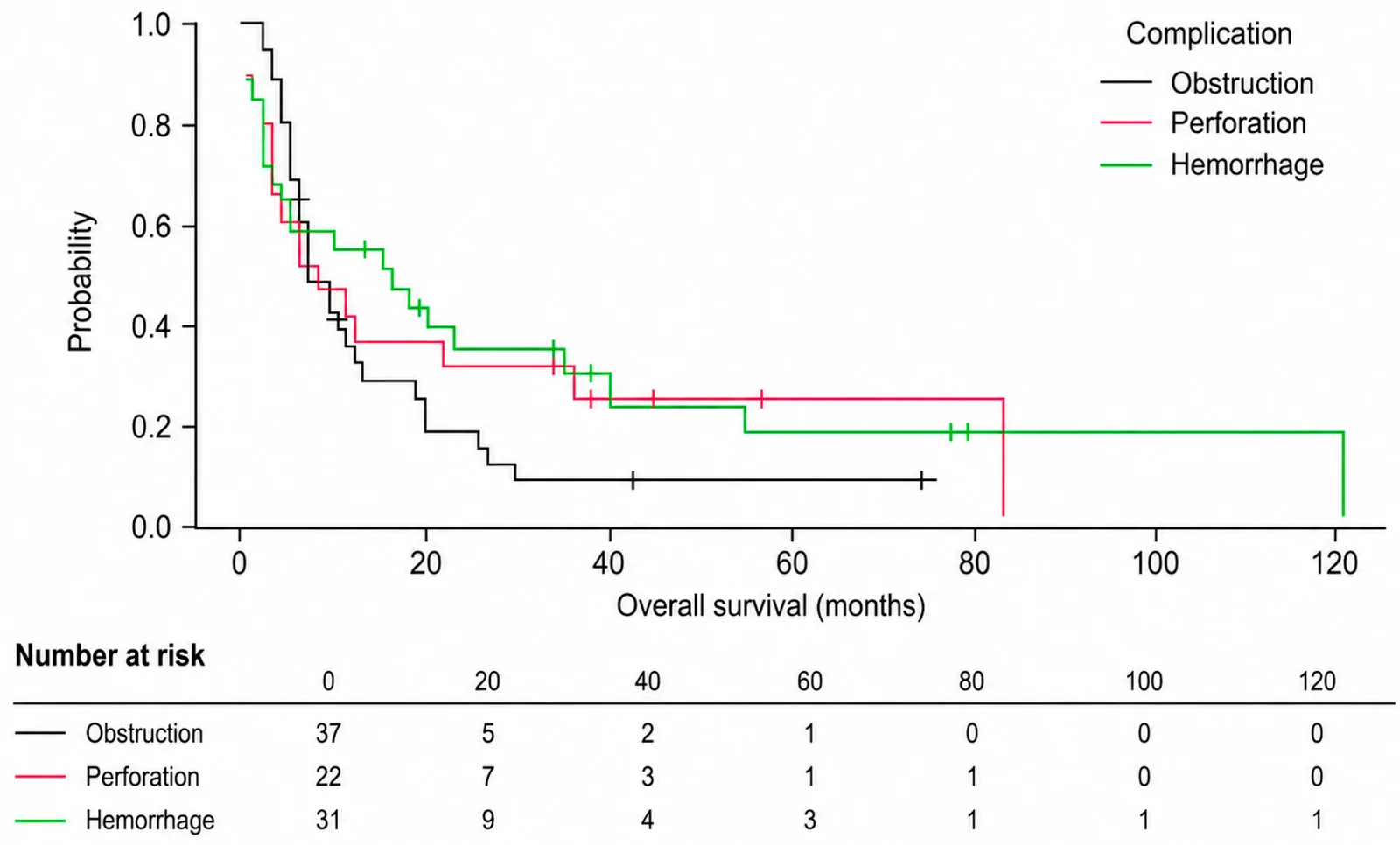
Kaplan–Meier curve of OS according to the type of diagnosis at the emergency department admission (black line: obstruction; red line: perforation; green line: hemorrhage; *p* = 0.50).

Kaplan–Meier survival analysis (Figure 2) did not reveal a statistically significant difference in overall survival between patients stratified according to the diagnosis at admission to the department (*p* = 0.50).

### 3.4. Factors Associated with the Type of Surgical Strategy

The palliative group’s mean age was significantly lower (63.7 vs. 73.4, *p* < 0.001) and the median BMI was higher (25 kg/m^2^ vs. 22.24 kg/m^2^, *p* = 0.01). Patients undergoing radical surgery had significantly fewer deaths at follow-up (75.4% vs. 100%, *p* = 0.014). There was no significant difference between groups for LOS (*p* = 0.083), neoadjuvant therapy (*p* = 0.79), 30-day mortality (*p* = 0.76), and initiation of adjuvant treatment (*p* = 0.26). These findings are summarized in Supplementary Table S3.

**Figure 3:**
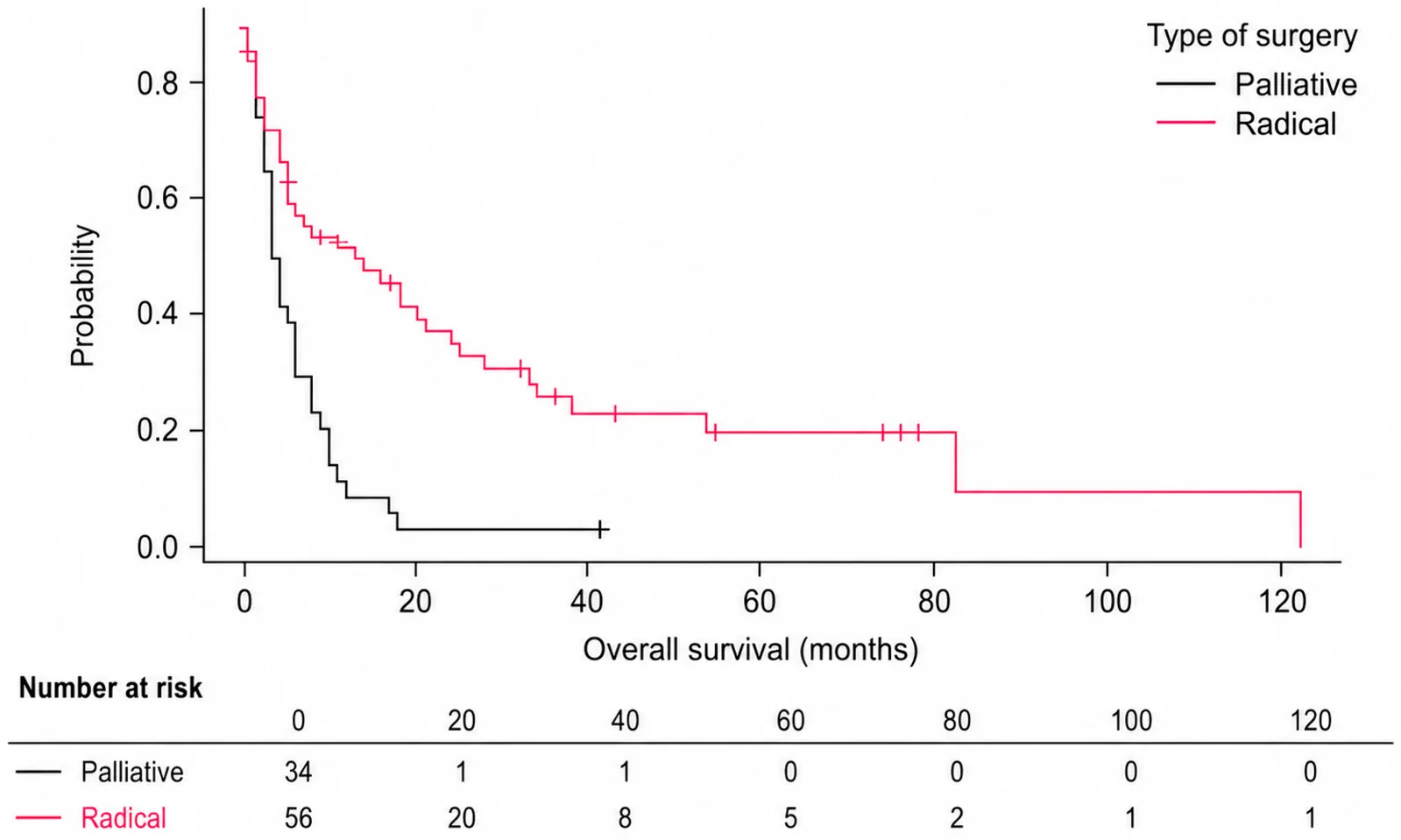
Kaplan–Meier curve of OS according to the type of surgical strategy (black line: palliative surgery; red line: radical surgery; *p* < 0.001).

Kaplan–Meier survival analysis (Figure 3) showed a statistically significant difference in OS between patients who underwent radical and palliative surgery (*p* < 0.001).

### 3.5. Univariate Analysis

In the univariate analysis (Table 2), age emerged as the only baseline factor significantly associated with perioperative mortality (*p* = 0.018), while not being associated with OS. Sex, BMI, and neoadjuvant therapy showed no significant correlation with either 30-day mortality or OS.

Histological subtype showed a trend toward significance, with intestinal-type tumors demonstrating longer OS (median 14 vs. 5.5 months, *p* = 0.07), without reaching conventional thresholds. Clinical presentation at ED (obstruction, hemorrhage, and perforation) did not influence either perioperative mortality risk (*p* = 0.569) or OS (*p* = 0.521). Conversely, surgical strategy had a significant prognostic impact: radical surgery was associated with improved survival compared to palliative procedures (median OS: 14 vs. 4.5 months, HR: 0.42, *p* < 0.001), despite having comparable 30-day mortality (*p* = 0.759).

### 3.6. Multivariable Analysis

The multivariate logistic regression model was built including the variables age, sex, BMI, surgical approach (gastrectomy vs. palliative procedure), histological subtype (diffuse vs. intestinal), and clinical presentation at admission.

In the full model, increasing age was associated with 30-day mortality (OR: 1.09, 95% CI: 1.02–1.17, *p* = 0.017), while diffuse histology showed a non-significant association with increased mortality and a wide confidence interval (OR: 4.92, 95% CI: 0.76–31.8, *p* = 0.094). Other covariates were not significantly associated with this outcome. After stepwise reduction, the most parsimonious model retained age and histology. Older age remained associated with higher 30-day mortality (OR: 1.09, 95% CI: 1.02–1.16, *p* = 0.011), while diffuse histology was also associated with higher mortality (OR: 5.85, 95% CI: 1.03–33.3, *p* = 0.046) (Table 3). The discriminatory performance of the final model was satisfactory, with an AUC of 0.782 (95% CI: 0.65–0.914) (Supplementary Figure S2).

Moreover, we conducted a Cox proportional hazards regression analysis to explore independent predictors of OS (Table 4). In the full Cox model, increasing age was associated with a higher risk of mortality (HR: 1.04, 95% CI: 1.01–1.06, *p* = 0.003), while undergoing gastrectomy (as opposed to palliative surgery) was associated with a lower hazard of death (HR: 0.32, 95% CI: 0.17–0.60, *p* < 0.001). Other covariates, including sex, BMI, histology, clinical urgency, and neoadjuvant therapy, were not significant. Stepwise selection by Akaike’s information criterion (AIC) confirmed that only age (HR: 1.03, 95% CI: 1.01–1.06, *p* = 0.005) and type of surgery (HR: 0.33, 95% CI: 0.19–0.55, *p* < 0.001) independently predicted OS in our cohort. The proportional hazards assumption was met for all variables.

## 4. Discussion

Emergency surgical interventions for patients with GC are often necessitated by life-threatening complications such as perforation, hemorrhage, or obstruction [4]. Despite substantial advancements in the management of GC, emergency surgery remains associated with significantly higher morbidity and mortality rates compared to elective procedures [4,19]. However, data regarding the outcomes of emergency gastric surgery are scarce in the current literature. In this exploratory study, we evaluated the 30-day mortality and OS after emergency GC surgery, exploring the factors that could influence survival, providing one of the largest multicenter Western series on emergency GC surgery. Results highlighted the challenges associated with these procedures, including high rates of postoperative complications, significant mortality, and the impact of histological type on long- term survival. In our cohort, severe complications (Clavien–Dindo grade ≥ 3) occurred in 28.8% of patients. The 30-day mortality (13.3%) and the global severe morbidity (28.8%) confirmed the high-risk profile of emergency GC surgery. These findings are consistent with previous studies, which have reported increased morbidity and mortality in emergency settings compared to elective procedures [4,7,8]. Havens et al. [7] found a mortality rate of 12.5% and a major complication rate of 32.8% in a large cohort of patients undergoing emergency general surgery, including gastric procedures. The authors identified emergency surgery as an independent risk factor for both complications (*p* = 0.001) and mortality (*p* = 0.029), emphasizing the inherent risks of operating on acutely ill patients with limited preoperative optimization. Similarly, a large retrospective study by Solsky et al. [4], which analyzed 9279 patients undergoing gastric surgery, found that those admitted through the emergency department had significantly higher mortality rates and longer LOS than elective cases.

These findings align with our results, suggesting that the physiological stress of acute complications, combined with the advanced stage of disease often seen in emergency presentations, contributes to poorer outcomes.

Nevertheless, despite the high prevalence of advanced gastric cancer in our cohort, worse outcomes may not be fully explained by advanced disease alone. Indeed, a study by Vasas et al. [8] found worse outcomes for GC patients presenting as emergencies when compared stage for stage with patients undergoing elective surgery. These results may rather indicate that patients who need emergency surgery are in an intrinsically poorer condition, regardless of the physical burden of the surgery, due to poorer nutritional status and limited preoperative optimization [9,20,21]. The relatively low rate of adjuvant chemotherapy (34%) may reflect postoperative frailty due to severe complications and early mortality, as described by Kasakura [19] and Hoshino [20]. Although 75.4% of patients achieved the recommended lymph-node yield, the average number remained inferior to that of elective series; the emergency setting, local inflammation, and haemodynamic instability are plausible explanations, similar to data from the Japanese National Clinical Database [20].

When analyzing the postoperative outcomes according to Lauren’s subtypes of GC, patients with diffuse-type GC had shorter OS compared to those with intestinal-type histopathology (5.5 months vs. 14 months), but it was not statistically significant (*p* = 0.07). However, these findings may highlight a potential association between diffuse-type histology and reduced OS. The lack of statistical significance might be due to limited statistical power. Further investigation with a larger sample size or adjustment for potential confounding factors is needed to confirm these findings. Furthermore, it is crucial to consider potential selection biases related to the classification of histologic type and whether this classification could be influenced by other prognostic factors. When analyzing perioperative mortality in a multivariable analysis, diffuse histopathology was associated with higher early mortality (*p* = 0.046) compared to intestinal type; however, cause-specific mortality data were not available. Moreover, the wide confidence interval indicates substantial uncertainty around the magnitude of this association, likely reflecting the limited number of 30-day deaths in our cohort. This finding should therefore be interpreted cautiously and requires validation in larger series. Our results are not consistent with the findings of Jimenez Fonseca et al. [13], who reported that, while diffuse-type GC had worse long-term survival, the short-term outcomes were not significantly different from intestinal-type GC.

In our cohort, diffuse-type GC was associated with a higher rate of positive surgical margins (29.6% vs. 6.7%, *p* = 0.03) and a greater number of positive lymph nodes (4.5 vs. 2, *p* = 0.016), which may contribute to the observed survival disparity. These findings are in line with the proven prognostic implications of Lauren’s classification [14,15,16,18], identifying diffuse-type GC as associated with poorer outcomes compared to intestinal-type GC, particularly in advanced stages, due to its propensity for early metastasis and resistance to conventional therapies.

Regarding the emergency clinical presentation, the most observed one in our cohort was gastric obstruction (41.1%), followed by hemorrhage (34.4%) and perforation (24.4%). We observed that patients undergoing surgery for hemorrhage were significantly older than those with obstruction (75.9 years vs. 65.4 years, *p* = 0.0014). This is consistent with the findings of Allum et al. [9], who noted that older patients are more likely to present with acute complications such as bleeding, which may be related to the use of anticoagulant therapy due to comorbidities.

Interestingly, we found no significant differences in OS based on the type of emergency presentation. This contrasts with the findings of Vasas et al. [8], who reported worse outcomes for patients presenting with perforation as compared to those with obstruction or hemorrhage, likely due to the higher risk of peritoneal contamination and sepsis. Conversely, our results align with those of Solsky et al. [4], who found no significant difference in survival based on the mode of emergency presentation. This discrepancy may be due to differences in the characteristics and size of the study populations.

We also observed a higher frequency of palliative surgery in patients presenting with obstruction, which may be attributed to the feasibility of managing the complication without resection, an approach that is less commonly viable in cases of hemorrhage and perforation.

As expected, our study showed a significant survival benefit associated with radical surgery as compared to palliative procedures (14 months vs. 4.5 months, *p* < 0.001). Patients who underwent radical surgery also had a lower rate of death at follow-up (75.4% vs. 100%, *p* = 0.014) and a 58% lower risk of death (HR: 0.42; 95% CI: 0.26–0.68; *p* < 0.001). Because surgical strategy was determined by resectability, disease burden, and clinical condition, this association is susceptible to confounding by indication and should not be interpreted as a causal treatment effect. These results are consistent with the findings of Markar et al. [10], who reported that curative resection for esophagogastric cancer had significantly better long-term survival as compared to palliative procedures, even in the emergency setting. The authors suggested that radical surgery should be considered whenever feasible, as it offers the best chance for long-term survival, particularly in patients with localized disease [3,10,22]. However, radical surgery was more frequently performed in patients with intestinal-type histology (88.2% vs. 46.4%, *p* < 0.001) in our cohort, which may have contributed to the observed survival benefit. This suggests that the feasibility of radical surgery is influenced by the histological subtype, likely due to differences in disease biology and the extent of peritoneal or distant metastases, or a different distribution of complications in the two Lauren’s types. In cases where radical surgery is not feasible, palliative procedures remain a crucial option for symptom management and improving quality of life [9].

Finally, while outcomes did not significantly differ between ACS and UGI units in our dataset, we believe in the importance of specialized expertise. Future studies may clarify whether outcomes improve when emergencies are managed by subspecialized teams.

Our study contributes to the scarce literature regarding emergency gastric surgery for GC in Western countries [23], thanks to the remarkable size of the sample and the multicentric design.

However, it has several limitations. First, the retrospective nature of the analysis may have introduced potential biases in patient selection and data collection. Second, the relatively small sample size (n = 90) may limit the statistical power to detect significant differences, particularly in subgroup analyses, which should be interpreted as exploratory. In addition, the small number of 30-day deaths limited the number of events available for multivariable logistic regression and may have resulted in model overfitting and imprecise effect estimates. Moreover, the lack of a control group undergoing elective surgery limits our ability to directly compare outcomes between emergency and elective settings. A major limitation is also the heterogeneity of surgical procedures—from palliative bypass to total gastrectomy—which hampers homogeneous comparisons of oncological outcomes, echoing findings by Collins et al. [24]. Finally, the comparison between radical and palliative surgery is subject to confounding by indication, because treatment selection depended on resectability, disease burden, and clinical condition, variables that could not be fully adjusted for in the present retrospective dataset. Unfortunately, standardized measures of acute physiological severity, comorbidity burden, anemia, nutritional status beyond BMI, anesthetic risk, and intensive-care requirement were not consistently available across the entire retrospective cohort. Residual confounding by baseline clinical severity therefore remains possible, particularly in the 30-day mortality analysis.

## 5. Conclusions

This study confirmed high morbidity and mortality for emergency gastric cancer surgery. Advanced age and diffuse histopathology were associated with higher 30-day mortality in our cohort. Radical surgery, when feasible, provided the best outcomes in terms of OS. The type of acute presentation did not significantly influence 30-day mortality and survival. These findings contribute to underscoring the importance of early diagnosis, careful perioperative planning, and specialized surgical expertise. Prospective multicenter studies in Western populations are warranted to refine patient selection and optimize outcomes.

## Figures and Tables

**Table 1:** Demographics and clinical characteristics of included patients.

Variable	All Patients (N = 90)
Age, years, mean (SD)	69.73 (12.61)
Female sex, No. (%)	36 (40)
BMI, median (IQR)	23.73 (21.22–25.96)
Neoadjuvant treatment, No. (%)	18 (20)
Diagnosis at the emergency department admission, No. (%)	
Perforation	22 (24.44)
Obstruction	37 (41.12)
Hemorrhage	31 (34.44)
Surgeon specialty, No. (%):	
Acute Care	29 (32.3)
Upper GI	61 (67.7)
Type of surgical strategy, No. (%)	
Radical	57 (63.3)
Palliative	33 (36.7)
Type of surgical treatment, No. (%)	
Subtotal gastrectomy	22 (24.5)
Total gastrectomy	35 (38.9)
Gastrostomy	4 (4.4)
Jejunostomy	18 (20)
Gastrointestinal bypass	11 (12.2)
Surgical approach, No. (%)	
Open	90 (100)
Laparoscopic	0 (0)
Postoperative complications ≥ 1, No. (%)	89 (98.9)
Clavien–Dindo classification, No. (%)	
0	1 (1.1)
1	42 (46.7)
2	21 (23.4)
3a	3 (3.3)
3b	9 (10)
4a	2 (2.2)
4b	3 (3.3)
5	9 (10)
Hospital stay, days, mean (IQR)	12.00 (8–20)
30-day mortality, No. (%)	12 (13.3)
Histological type	
Intestinal	34 (37.8)
Diffuse	56 (62.2)
Resection margin, No. (%) *	
Positive	10 (11.1)
Negative	47 (83.9)
T stage, No. (%) *	
1a	1 (1.8)
1b	8 (14.3)
2	11 (19.6)
3	12 (21.4)
4a	18 (32.1)
4b	5 (8.8)
N stage, No. (%) *	
N0	18 (32.2)
N1	11 (19.6)
N2	11 (19.6)
N3a	11 (19.6)
N3b	5 (9)
Harvested lymph nodes, mean (IQR) *	26 (17–32)
Harvested lymph nodes ≥ 16, No. (%) *	43 (75.4)
Positive harvested lymph nodes, mean (IQR) *	2.5 (0–7)
Initiation of adjuvant chemotherapy, No. (%)	31 (34.4)
Overall survival, months median (IQR)	6.5 (3–19)
Death at follow-up, No. (%)	76 (84.4)

Abbreviations: IQR, interquartile range; SD, standard deviation; upper GI, upper gastrointestinal. * Calculated from patients receiving radical surgical treatment.

**Table 2:** Univariate analysis for 30-day mortality and overall survival (OS).

Variable	30-Day Mortality	*p*	OS	*p*
**Age (Years)**	77.7 ± 10.3 vs. 68.5 ± 12.5	**0.018**	HR: 1.02 (95% CI: 0.99–1.04)	0.125
**Sex (Female vs. Male)**	19.4% vs. 9.3%	0.210	HR: 1.42 (95% CI: 0.89–2.26)	0.138
**BMI**	23.1 (20.7–24.6) vs. 24.2 (21.3–26.0)	0.413	HR: 0.98 (95% CI: 0.92–1.04)	0.433
**Neoadjuvant treatment**	11.1% vs. 13.9%	1.000	HR: 0.81 (95% CI: 0.44–1.48)	0.493
**Diagnosis at ED (Obstruction vs. Hemorrhage vs. Perforation)**	10.8% vs. 9.1% vs. 19.4%	0.569	Median OS: 7 vs. 6 vs. 9 mo	0.521
**Surgical strategy (Palliative vs. Radical)**	14.7% vs. 12.5%	0.759	Median OS: 4.5 vs. 14 mo; HR: 0.42 (95% CI: 0.26–0.68)	**<0.001**
**Histology (Intestinal vs. Diffuse)**	5.9% vs. 17.9%	0.124	Median OS: 14 vs. 5.5 mo	0.070

Abbreviations: BMI, body mass index; ED, emergency department; OS, overall survival.

**Table 3:** Multivariable analysis for 30-day mortality.

Variable	OR (Full Model)	95% CI	*p*	OR (Stepwise)	95% CI	*p*
**Age (per year)**	1.09	1.02–1.17	**0.017**	1.09	1.02–1.16	**0.011**
**Sex (male)**	1.73	0.41–7.28	0.455	–	–	–
**BMI**	0.98	0.80–1.19	0.812	–	–	–
**Gastrectomy**	0.68	0.14–3.40	0.639	–	–	–
**Histology (diffuse)**	4.92	0.76–31.8	0.094	5.85	1.03–33.3	**0.046**
ED presentation *	–	–	>0.7–0.8	–	–	–

* Baseline (0) = obstruction. Abbreviations: BMI, body mass index; ED, emergency department; OR, odds ratio.

**Table 4:** Multivariable analysis for overall survival (OS).

Variable	HR (Full Model)	95% CI	*p*	HR (Stepwise)	95% CI	*p*
**Age (per year)**	1.037	1.012–1.062	**0.003**	1.033	1.010–1.057	**0.005**
**Sex (male)**	1.209	0.709–2.060	0.487	–	–	–
**BMI**	0.974	0.911–1.042	0.439	–	–	–
**Gastrectomy**	0.316	0.166–0.600	**<0.001**	0.326	0.195–0.546	**<0.001**
**Histology (diffuse)**	1.110	0.637–1.932	0.713	–	–	–
ED presentation *	1.518/0.984	Perforation vs. obstruction HR = 1.52 (95% CI: 0.78–2.96)	0.221	–	–	–
		Hemorrhage vs. obstruction HR = 0.98 (95% CI: 0.49–1.97)	0.964			
**Neoadjuvant Therapy**	0.683	0.354–1.316	0.254	–	–	–

* Baseline (0) = obstruction. Abbreviations: HR, hazard ratio; BMI, body mass index.

## Data Availability

The data presented in this study are available on request from the corresponding author due to privacy reasons.

## Supplementary Materials

The following supporting information can be downloaded at: https://www.mdpi.com/article/10.3390/jcm15166395/s1, Table S1. Variables associated with the histological type (intestinal vs. diffuse); Table S2. Variables associated with the diagnosis at the Emergency Department admission (perforation vs. obstruction vs. hemorrhage); Table S3. Variables associated with the type of surgical strategy (radical vs. palliative); Figure S1. Flow chart of patients’ inclusion in the study; Figure S2. ROC curve for multivariable analysis; AUC = 0.782 (95% CI 0.65–0.914).
