# Supplementary material for: Gastric Cancer Surgery in an Emergency Setting: Insights from a Western Multicenter Cohort
Source: J Clin Med. 2026 Aug 19;15(16):6395. doi: 10.3390/jcm15166395 (PMC13514142; doi:10.3390/jcm15166395)

Supplementary Table S1. Variables associated with the histological type (intestinal vs. diffuse).

**Variable**	**Intestinal type (N=34)**	**Diffuse type (N=56)**	***P* value**
Age, years, mean (SD)	72.32 (12.49)	68.16 (12.53)	0.13
Female sex, No. (%)	8 (23.5)	28 (50)	**0.01**
BMI, median (IQR)	24.92 (22.58-26.1)	23.82 (20.81-25.73)	0.086
Neoadjuvant treatment, No. (%)	8 (23.5)	10 (17.9)	0.59
Type of surgical strategy, No. (%)			
Radical	30 (88.2)	27 (48.2)	**<0.001**
Palliative	4 (11.8)	29 (51.8)	
Hospital stay, days, median (IQR)	15 (10.5-26.5)	11 (7.5-16.5)	**0.01**
30-day mortality, No. (%)	2 (5.9)	10 (17.85)	0.124
Resection margin, No. (%)*			
Positive	2 (6.7)	8 (29.6)	**0.03**
Negative	28 (93.3)	19 (70.4)	
Harvested lymph nodes, median (IQR)*	23 (19-35)	27 (12.5-27.5)	0.169
Harvested lymph nodes ≥16, No. (%)*	24 (80)	19 (73.1)	0.54
Positive harvested lymph nodes, median (IQR)*	2 (0-5.75)	4.5 (1.25-14.5)	**0.016**
Initiation of adjuvant chemotherapy, No. (%)	14 (41.2)	17 (25)	0.36
Overall survival, months, median (IQR)	14 (5-32)	5.5 (2-11)	**0.015**
Death at follow-up, No. (%)	29 (85.3)	47 (83.9)	1.00

Statistically significant results are highlighted in bold. Abbreviations: CI, confidential interval; IQR, interquartile range; OR, odds ratio; SD, standard deviation.
*Calculated from 57 patients receiving a radical surgical treatment.

Supplementary Table S2. Variables associated with the diagnosis at the Emergency Department admission (perforation vs. obstruction vs. hemorrhage).

**Variable**	**Perforation (N=22)**	**Obstruction (N=37)**	**Hemorrhage (N=31)**	***P* value**
Age, years, mean (SD)	68.45 (13.20)	65.35 (11.72)	75.87 (11.01)	**0.002**
Female sex, No. (%)	7 (15.56)	14 (7.78)	15 (16.67)	0.46
BMI, median	24.89	22.66	24.10	0.32
Neoadjuvant treatment, No. (%)	8 (8.89)	6 (6.67)	4 (4.44)	0.1
Type of surgical strategy, No. (%)				
Radical	17 (18.89)	14 (15.56)	25 (28.78)	**<0.001**
Palliative	5 (5.56)	23 (25.56)	6 (6.67)	
Hospital stay, days, median	18.5	12	12	0.1
30-day mortality, No. (%)	2 (2.22)	4 (4.44)	6 (6.67)	0.57
Resection margin, No. (%)*				
Positive	2 (3.5)	5 (8.8)	3 (5.3)	0.1
Negative	16 (28.1)	9 (15.8)	22 (38.6)	
Harvested lymph nodes, median (IQR)*	27 (19-32)	24 (13-40)	24 (15-30)	0.60
Harvested lymph nodes ≥16, No. (%)*	15 (26.8)	9 (16.1)	19 (33.9)	0.28
Positive harvested lymph nodes, median (IQR)*	4 (1-7)	5 (1-8)	2 (0-5)	0.33
Initiation of adjuvant chemotherapy, No. (%)	11 (12.2)	10 (11.1)	10 (11.1)	0.34
Overall survival, months, median (IQR)	6 (2-21)	7 (5-10)	9 (2-22)	0.98
Death at follow-up, No. (%)	18 (20)	33 (36.7)	25 (27.8)	0.58

Statistically significant results are highlighted in bold. Abbreviations: IQR, interquartile range; SD, standard deviation.
*Calculated from 57 patients receiving a radical surgical treatment.

Supplementary Table S3. Variables associated with the type of surgical strategy (radical vs. palliative).

**Variable**	**Radical surgery (N=57)**	**Palliative surgery (N=33)**	***P* value**
Age, years, mean (SD)	73.39 (11.38)	63.71 (12.36)	**<0.001**
Female sex, No. (%)	19 (33.33)	17 (51.5)	0.18
BMI, median (IQR)	25 (22.21-26.46)	22.24 (20.21-24.61)	**0.01**
Neoadjuvant treatment, No. (%)	12 (21.1)	6 (18.2)	0.79
Hospital stay, days, median (IQR)	13 (9-21.5)	12 (8-15)	0.083
30-day mortality, No. (%)	7 (12.3)	5 (15.15)	0.76
Initiation of adjuvant chemotherapy, No. (%)	22 (38.6)	9 (27.3)	0.26
Overall survival, months, median (IQR)	14 (6-25)	4.5 (3-7)	**0.013**
Death at follow-up, No. (%)	43 (75.4)	33 (100)	**0.014**

Statistically significant results are highlighted in bold. Abbreviations: CI, confidential interval; IQR, interquartile range; OR, odds ratio; SD, standard deviation.

Supplementary Figure S1. Flow chart of patients' inclusion in the study.

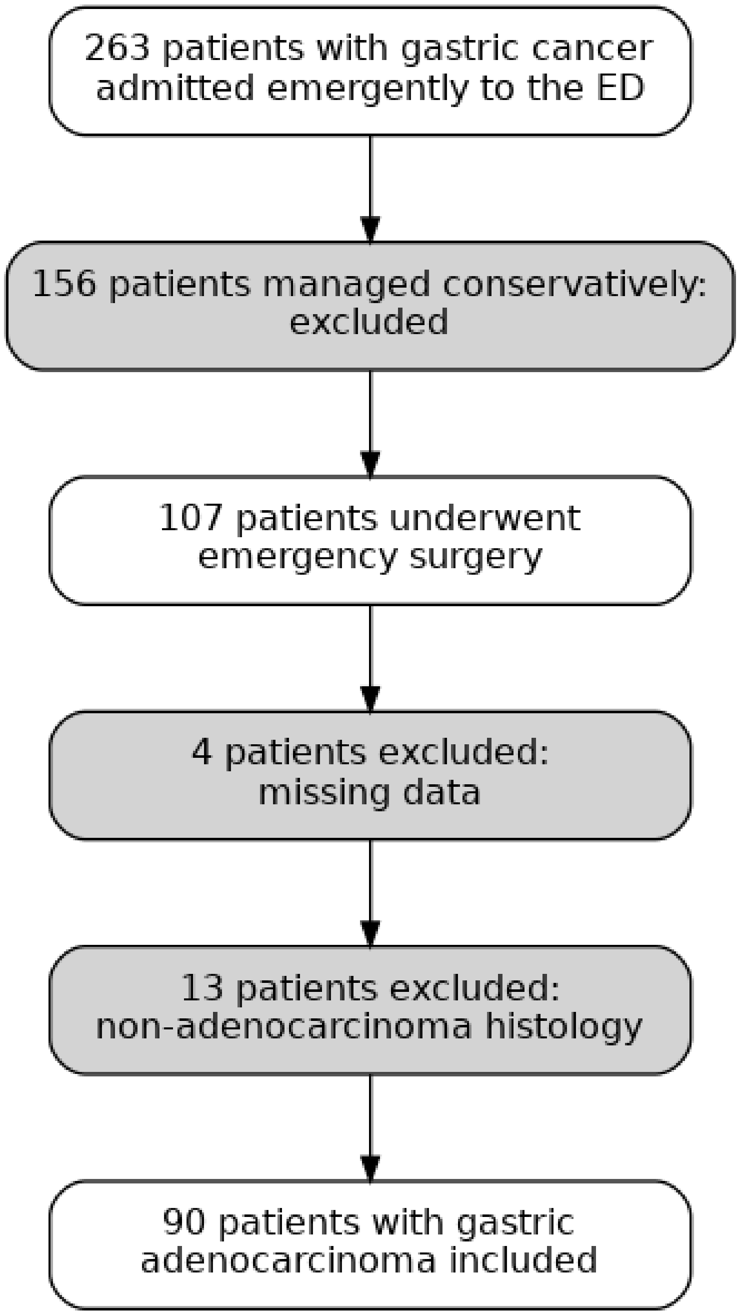

Supplementary Figure S2. ROC curve for multivariable analysis; AUC= 0.782 (95% CI 0.65-0.914).

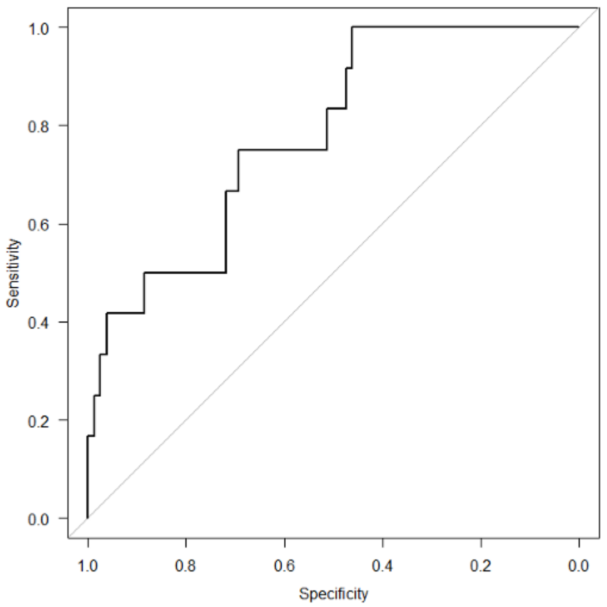

Supplement: Supplementary file 1 [file jcm-15-06395-s001.zip › jcm-4469039-supplementary.pdf]
